# Development of a clinical version of the Carers’ Needs Assessment for Schizophrenia

**DOI:** 10.1007/s40211-017-0240-3

**Published:** 2017-08-08

**Authors:** Johannes Wancata, Fabian Friedrich, Annemarie Unger, Rebecca Jahn

**Affiliations:** Clinical Division of Social Psychiatry, Department of Psychiatry and Psychotherapy, Medical University of Vienna, Währinger Gürtel 18–20, 1090 Vienna, Austria

**Keywords:** Schizophrenia, Caregivers, Needs, Feasibility, Schizophrenie, Angehörige, Bedarf, Machbarkeit

## Abstract

**Objective:**

Based on the research version of the Carers’ Needs Assessment for Schizophrenia (CNA-S) a shortened clinical version was developed for routine assessment of interventions needed by the caregivers of schizophrenia patients in everyday clinical work.

**Methods:**

The development of this questionnaire (including a manual explaining its use) was based on results from earlier studies and suggestions from Austrian researchers involved in previous studies using the research version. Based on discussions with researchers the questionnaire and the manual were improved step by step. A clinical test version was investigated for feasibility and practicability in two waves of 15 caregivers of schizophrenia patients each.

**Results:**

More than 90% of caregivers perceived the clinical version of the CNA-S as a useful instrument to assess all relevant aspects of caregivers’ needs and problems. They reported feeling well during the interview for the CNA-S. Clinicians using the clinical version of the CNA-S reported similar views.

**Conclusion:**

These results suggest that the clinical version of the CNA-S is both feasible and practicable in everyday clinical work.

## Zusammenfassung

**Ziel:**

Auf Basis der Forschungsversion des „Carers’ Needs Assessment for Schizophrenia (CNA-S)“ wurde eine verkürzte und vereinfachte klinische Version entwickelt. Diese soll für die Routineerhebung des Bedarfs von Angehörigen Schizophrenie-Kranker geeignet sein.

**Methodik:**

Dieser Fragebogen sowie ein erläuterndes Manual wurden aufgrund der Daten früherer Studien sowie der Empfehlungen von klinisch tätigen Wissenschaftlern entwickelt, die früher die Forschungsversion für Studien verwendet hatten. In weiteren Diskussionen mit diesen Wissenschaftlern wurden Fragebogen und Manual in mehreren Schritten verbessert. Eine klinische Testversion wurde auf Machbarkeit und Praktikabilität geprüft, wobei 2 Gruppen zu je 15 Angehören befragt wurden.

**Ergebnisse:**

Mehr als 90 % der Angehörigen schätzten die klinische Version des CNA-S als hilfreich ein, um alle relevanten Problembereiche von Angehörigen zu erfassen. Ebenfalls mehr als 90 % der Angehörigen gaben an, sich während des klinischen Interviews zur Erfassung ihres Bedarfs wohlgefühlt zu haben. Ähnliche Ergebnisse wurden von den klinisch Tätigen über die Verwendung der klinischen Version des CNA-S berichtet.

**Schlussfolgerungen:**

Die Ergebnisse weisen darauf hin, dass dieser Fragebogen sowohl praktikabel als auch klinisch gut verwendbar ist.

## Introduction

Numerous studies reported negative consequences of schizophrenia on family caregivers such as stress, reduced social contacts and emotional burden [1–5]. Further, caregivers of schizophrenia patients show an increased risk for depression [6, 7].

Other studies revealed that interventions to support caregivers and to improve their communication with the patient are effective in terms of reduced relapses or fewer hospital admissions [8, 9]. Therefore, interventions for family caregivers are well established as an integral part of standard care of schizophrenia patients [10].

For service planning, it is necessary to know which interventions are needed and how frequently they should be applied [11]. Thus, an instrument to assess the needs of caregivers of schizophrenia patients (Carers’ Needs Assessment for Schizophrenia, CNA-S) was developed. It was designed as a research instrument based on a “bottom-up” approach [12]. According to van Haaster et al. [13] needs can be assessed on three levels: first, the problems experienced by the patient; second, the interventions required to alleviate or solve these problems; third, the services required to provide these interventions. The CNA-S assesses needs on the intervention level, after assessing which problems are present. Psychometric properties of the CNA-S such as content validity, concurrent validity, test–retest reliability and interrater reliability are sufficiently good (for details see Wancata et al. 2006 [12]).

In the last 10 years, the CNA-S has spread and schizophrenia researchers worldwide (e. g. United Kingdom, Germany, India and Greece) have been using it [14–16]. The CNA-S also raised interest among clinicians calling for a clinical version to use in the treatment of schizophrenia patients and their relatives.

Thus, the objective of the present study was to develop an assessment instrument for the needs of schizophrenia patients’ caregivers meeting the requirements for the use in clinical practice. This questionnaire should help clinicians to easily identify caregivers’ problems and the needed interventions. In order to support clinical work, the questionnaire has to be feasible and time-effective posing as little burden for the caregivers as possible. In addition, it should be easy to use, requiring no comprehensive training. Results should be presented clearly to prevent the clinician from overlooking important aspects. Therefore, a checklist format seemed to be most appropriate.

Since the research version of the CNA-S is comprehensive and its psychometric properties are sufficiently good [12], the clinical version should be based on the content of this research instrument.

## Methods

### Development of the clinical version of the CNA-S

First, CNA-S datasets of 393 caregivers deriving from three different Austrian studies having used the research version of the CNA-S were analysed to identify items rarely used (Examples of these analyses are available on request from the first author.).

This information was provided to several Austrian researchers having used the research version of the CNA-S in previous studies. They were then asked to make suggestions on how the CNA-S can be shortened or made easier without loss of relevant information for clinical purposes. They made the following recommendations:

- Exclude problems and interventions which are not common.
- Combine some problem areas and some interventions to make the assessment more easy to use in everyday work.
- Differentiate between urgent interventions and those which should be offered later.
- Rank interventions by urgency.
- A checklist should include the opportunity to make notes about practical information (e. g. phone numbers, contact persons).
- A checklist usually gives information about the present situation of the caregiver. After some months an update of the needed interventions might be necessary. This option should be considered.

Frequency analyses and suggestions from researchers of previous studies (psychiatrists as well as psychologists) were used to develop the first version of the clinical CNA-S. Since this version should not require any comprehensive training for its application, a short instruction manual (including two case vignettes) was developed. The manual as well as the first version of the questionnaire were discussed with the researchers mentioned above who were also experienced in clinical work with schizophrenia patients and their families. Based on these discussions the questionnaire and the manual were improved step by step (modified Delphi process [17]). Finally, the 5th version reached saturation and was the first clinical test version.

### Structure and content of the clinical version

The clinical version of the CNA-S consists of a questionnaire structured like a checklist and a short manual explaining its use. The questionnaire includes 13 problem areas. For each of these problem areas the questionnaire offers several possible interventions (between two and six interventions per problem area, details are given in the results section). The same interventions (e. g. individual counselling) might be helpful for solving different problems (e. g. conflicts with the patient, feelings of guilt, not enough time). Therefore, some interventions occur in more than one problem area of this instrument. Both the list of problem areas as well as the list of possible interventions should serve as a guide in everyday clinical work to avoid overlooking important problems of the caregivers which might also affect the patient.

After a short interview with the caregiver, the questionnaire is filled out by the interviewer based on their clinical judgement. This is likely to result in several interventions which might be useful for a specific caregiver. In a next step the clinician has to decide which interventions are most important and should be administered first. Therefore, an extra form is provided where the clinician can rank the interventions by urgency (The clinical version of the CNA-S will be freely available to every psychiatric professional.).

### Feasibility testing of the clinical test version

Adult family caregivers (18+ years, both genders) of adult patients (18+ years, both genders) suffering from schizophrenia according to ICD-10 (based on routine clinical assessments) and patients were invited to participate in this study. Caregivers (usually the person who has the most contact to the patient according to the patient) were included only with the patient’s consent. In order to include caregivers of patients in different stages of the illness we identified them from three different sources: outpatients, inpatients and day hospital patients of the Clinical Division of Social Psychiatry of the Medical University of Vienna. It was intended to include about 15 caregivers in each wave of feasibility testing.

After applying the clinical version of the CNA-S interviewers and caregivers answered questions regarding its practicability, clarity and feasibility in clinical work. A small set of closed-ended as well as open-ended questions were used. This part of the study was performed by staff not involved in previous parts of the study. They were specialists in psychiatry who treated the patients routinely at the Clinical Division of Social Psychiatry of the Medical University of Vienna. The results (especially responses to open-ended questions) were used to improve the content, the wording and the formatting of the first clinical test version, resulting in a second one. It was intended to repeat this process described above until a clinical test version achieves at least 75% favourable ratings by participating caregivers and interviewers.

Informed consent from the patients *and* their caregivers was obtained. The study was approved by the Ethics Committee of the Medical University of Vienna.

## Results

### Feasibility testing of the first clinical test version

The first clinical test version was used among 16 caregivers (56.3% female, mean age 50.1 years, standard deviation [SD] 10.9 years) of schizophrenia patients (25.0% female, mean age 23.3 years, SD 3.2 years). The mean time needed to perform the interviews and to fill out the questionnaires was 28.1 min (SD 15.0 min).

Caregivers as well as interviewers participating in the first test wave made several critical comments and suggestions on how to improve the questionnaire. A relevant proportion of caregivers and interviewers rated the questionnaire as “difficult to use” or as a relevant burden to caregivers. Based on these comments, the wording of several parts of the questionnaire was adapted.

### Feasibility testing of the second clinical test version

This test version was used among 15 caregivers (60% female; mean age 44.9 years, SD 14.7 years) of schizophrenia patients. Caregivers were predominantly mothers (53.3%) followed by fathers (13.3%), siblings (6.7%), partners (6.7%) and other family members (20.0%). Nearly half of the caregivers (46.7%) lived in the same household as the patient and two thirds had more than 10 h per week of personal contact with the patient. About a quarter of caregivers were married, about a quarter divorced and 40% were single.

More than half of the patients were female (53.3%), and their mean age was 29.3 years (SD 9.3 years). Three quarters of patients were single and one quarter was divorced (nobody married or widowed). Their mean duration of illness was 7.4 years (SD 4.2 years). In all, 46.7% were day hospital patients, 33.3% were inpatients and 20.0% were outpatients.

In the second wave, the mean time needed to perform the interviews and to fill out the questionnaires was 22.5 min (SD 8.4 min).

More than 90% of caregivers perceived the second clinical test version of the CNA-S as a useful instrument to assess all relevant aspects of caregivers’ needs and problems (Table 1). They reported feeling well during the interview. In addition, 86.7% of interviewers had the impression that using the CNA-S is no or only a small burden to the caregivers and all of them reported that the clinical version of the CNA-S was easy or very easy to use. Concerning open-ended questions only one suggestion of improvement was made (6.7% of all interviews of the second wave), which was about providing more response categories but not giving any proposals. A total of 93.3% of all interviews of the second wave indicated no need for clarification or improvement. Thus, the second clinical test version (overall 6th version) achieved sufficient acceptability and usefulness exceeding the stop criterion of 75% favourable ratings. Thus, no more changes of were made.

**Table 1:** Responses of second (final) caregiver sample feasibility testing (dichotomized)

*Questions to caregivers*		
Do you think this questionnaire is useful in order to assess all relevant aspects of the caregiver?	Very much or moderate (in %)	93.3
	Little or not (in %)	6.7
Did you feel well during our conversation when interviewer used this questionnaire?	Very or rather well (in %)	93.3
	Rather not well or not well (in %)	6.7
The questionnaire was helpful when interviewing the patient?	Very much or moderate (in %)	93.3
	Little or not (in %)	6.7
*Questions to interviewers*		
How was the use of the questionnaire?	Difficult to use (in %)	0.0
	Easy or very easy to use (in %)	100.0
The caregiver seemed to be burdened by performing an interview based on this questionnaire	No or small burden (in %)	86.7
	Moderate or large burden (in %)	13.3

### Frequencies of problems and needs in the second clinical test version

Table 2 shows all problem areas of the clinical version and gives the frequencies reported during the of final feasibility testing. The most frequent problems were “Not enough information about illness, its course and symptoms” (66.7%) and “Burden due to past incidents, concerns because of chronic course of illness” (66.7%), followed by “Concerns due to patient’s lack of reasonability” (53.3%) and “Not enough information about treatment and rehabilitation” (53.3%).

**Table 2:** Frequency of caregivers’ problems of final feasibility testing

Number	Problem areas	%
1	Not enough information about illness, its course and symptoms	66.7
2	Not enough information about treatment and rehabilitation	53.3
3	Not enough information about relapses and how to manage crises	46.7
4	Fear of stigma, insufficient knowledge about psychiatric services	13.3
5	Difficulties communicating and conflicts with the patient	33.3
6	Concerns due to patient’s lack of reasonability, critical situations at home during last few months	53.3
7	Burden due to past incidents (e. g. aggressions), concerns because of chronic course of illness	66.7
8	Financial burden because of patient, insufficient information about patient’s financial entitlements	46.7
9	Feelings of guilt of the patient’s condition, being blamed by others	6.7
10	Not enough time, help by others caring for the patient	26.7
11	Lack of contact with other people, tense situations in the family	13.3
12	Patient lives or stays frequently in the carer’s household, difficulties resulting from this	20.0
13	Carer feels exhausted or sick	33.3

Interventions needed for each problem area are shown in Table 3. For example, of those having a problem from problem area 1 (“Not enough information about illness, its course and symptoms”) nearly half of all caregivers needed “Individual psychoeducation” and “Printed information material”, while only 13.3% needed “Group psychoeducation”.

**Table 3:** Frequency of needed interventions (per problem area) of final caregivers’ feasibility testing

Problem number	Intervention number	Interventions	%
1	A	Individual psychoeducation	46.7
	B	Group psychoeducation	13.3
	C	Printed information material	46.7
2	A	Individual psychoeducation	60.0
	B	Group psychoeducation	6.7
	C	Printed information material	20.0
3	A	Individual psychoeducation	40.0
	F	Individual counselling and support	0.0
	B	Group psychoeducation	13.3
	C	Printed information material	20.0
4	A	Individual psychoeducation	0.0
	F	Individual counselling and support	6.7
	D	Self-help group for family members	13.3
	E	Relatives group guided by a professional	0.0
5	F	Individual counselling and support	20.0
	G	Family counselling	20.0
	E	Relatives group guided by a professional	6.7
	M	Separate accommodation for the patient	6.7
	J	Establishment of a daily program for the patients	33.3
	I	Case management for patient	6.7
6	P	Crisis service (phone, mobile, including home visits)	0.0
	L	Temporary supervision of the patient at home by a professional	13.3
	F	Individual counselling and support	20.0
	I	Case management for patient	26.7
	D	Self-help group for family members	0.0
	E	Relatives group guided by a professional	13.3
7	F	Individual counselling and support	46.7
	G	Family counselling	40.0
	D	Self-help group for family members	6.7
	E	Relatives group guided by a professional	20.0
8	H	Counselling by a social worker	40.0
	K	Financial support	33.3
9	F	Individual counselling and support	6.7
	G	Family counselling	6.7
	B	Group psychoeducation	0.0
	D	Self-help group for family members	0.0
	E	Relatives group guided by a professional	0.0
10	F	Individual counselling and support	0.0
	J	Establishment of a daily program for the patients	26.7
	L	Temporary supervision of the patient at home by a professional	13.3
	Q	Holidays organized specifically for patients	6.7
11	D	Self-help group for family members	0.0
	E	Relatives group guided by a professional	0.0
	F	Individual counselling and support	6.7
	N	Psychotherapy, Family therapy	6.7
12	H	Counselling by a social worker	0.0
	M	Separate accommodation for the patient	0.0
	F	Individual counselling and support	20.0
13	F	Individual counselling and support	6.7
	N	Psychotherapy, Family therapy	20.0
	O	Diagnosis and/or treatment of the carer by a general practitioner	26.7

Some interventions might be helpful for solving different problems. Accordingly, they occur in more than one problem area and are rated separately for each problem area. For clinical purposes (i. e. providing interventions to caregivers) it would be redundant to plan identical interventions for each problem area separately. Therefore, it is necessary to know how often an intervention is needed overall.

Thus, the overall frequencies of needed interventions merged from all problem areas are shown in Table 4. Most frequently needed interventions overall were “Individual counselling and support” (80%) and “Individual psychoeducation” (66.7%).

**Table 4:** Frequency of needed interventions (overall frequency) of final caregivers’ feasibility testing

Intervention number	Interventions	%
A	Individual psychoeducation	66.7
B	Group psychoeducation	20.0
C	Printed information material	46.7
D	Self-help group for family members	20.0
E	Relatives group guided by a professional	26.7
F	Individual counselling and support	80.0
G	Family counselling	60.0
H	Counselling by a social worker	40.0
I	Case management for patient	33.3
J	Establishment of a daily program for the patients	46.7
K	Financial support	33.3
L	Temporary supervision of the patient at home by a professional	20.0
M	Separate accommodation for the patient	6.7
N	Psychotherapy, Family therapy	20.0
O	Diagnosis and/or treatment of the carer by a general practitioner	26.7
P	Crisis service (phone, mobile, including home visits)	0.0
Q	Holidays organized specifically for patients	6.7

## Discussion

Overall, the attempt to develop a straightforward version for clinical use of the comprehensive research instrument CNA-S was successful. The development was based on the frequency of problems and necessary interventions reported in previous Austrian studies having used the CNA-S. Feasibility and practicability of the final clinical version were satisfactory. The participating caregivers found the questionnaire useful and it was not perceived as a burden. The clinical version of the CNA-S proved to be time-effective with completion of the interview and filling out the questionnaire in less than 30 min which makes it a practical and easy instrument for everyday clinical work.

The restriction to Austrian data may be perceived as a limitation because the generalizability to other countries could be impaired. Nevertheless, we used data from nearly 400 caregivers of three independent studies [6, 7, 18] including inpatients, outpatients and day hospital patients. Data of these studies were collected in university services and in nonuniversity psychiatric services deriving from five Austrian provinces. This ensures variability of the samples reducing the risk of selection bias.

The questionnaire and the manual were improved step by step in discussions with psychiatric and psychological experts who had used the research version before. This procedure needed five versions until saturation was yielded. Thus, the 5th version was the first clinical test version. Regarding feasibility and practicability the second test version reached the predetermined stop criterion for further improvement. We were surprised that only two clinical test versions were necessary to reach such high rates of positive responses. These high rates were yielded even though all participating caregivers and some interviewers differed between the two waves of testing. The sample size of 15 persons per test wave may seem rather small, but is in agreement with earlier studies [19].

The clinical version of the CNA-S developed in this study was derived from the research instrument which was tested for content and concurrent validity. Thus, a larger multicentre study is planned comparing the two versions, whereas high agreement could serve as an indicator of content validity of the clinical version. In addition, investigations of reliability are planned. We hope that this straightforward and practical questionnaire can help to identify and specify problems and needs of caregivers and to provide the right interventions in everyday clinical work. Thus, it could have a positive impact on the recovery process of patients (The clinical CNA-S is available from the first author.).
